# Percutaneous treatment of chest wall chondroid hamartomas: the experience of a single center

**DOI:** 10.1007/s00247-022-05498-1

**Published:** 2022-09-05

**Authors:** Alessandro Inserra, Cristina Martucci, Giulia Cassanelli, Alessandro Crocoli, Guglielmo Paolantonio, Lorenzo M. Gregori, Gian Luigi Natali

**Affiliations:** 1 General and Thoracic Surgery Unit, Department of Pediatric Surgery, Bambino Gesù Children’s Hospital, IRCCS, Piazza Sant’Onofrio 4, 00165 Rome, RM Italy; 2 Interventional Radiology Unit, Department of Imaging, Bambino Gesù Children’s Hospital, IRCCS, Rome, Italy; 3 Surgical Oncology Unit, Department of Pediatric Surgery, Bambino Gesù Children’s Hospital, IRCCS, Rome, Italy

**Keywords:** Chest wall, Children, Computed tomography, Cryosurgery, Hamartoma, Microwave, Radiofrequency ablation, Ribs, Thorax

## Abstract

**Background:**

Thoracic mesenchymal hamartomas are rare benign lesions. Rarely symptomatic, they may compress pulmonary parenchyma, leading to respiratory distress. Although spontaneous regression has been documented, the more common outcome is progressive growth. The treatment of choice is en bloc excision of the involved portion of the chest wall, frequently leading to significant deformity.

**Objective:**

The aim of our study was to describe percutaneous techniques to treat these lesions.

**Materials and methods:**

We collected data of children with thoracic mesenchymal hamartomas who were treated at our institution from 2005 to 2020 using various percutaneous techniques. Techniques included radiofrequency thermoablation, microwave thermoablation (microwave thermoablation) and cryoablation.

**Results:**

Five children were treated for chest wall hamartomas; one child showed bilateral localization of the mass. Two children underwent microwave thermoablation, one radiofrequency thermoablation and two cryoablation; one child treated with cryoablation also had radiofrequency thermoablation because mass volume increased after the cryoablation procedure. The median reduction of tumor volume was 69.6% (24.0–96.5%). One child treated with microwave thermoablation showed volumetric increase of the mass and underwent surgical removal of the tumor. No major complication was reported.

**Conclusion:**

Percutaneous ablation is technically feasible for expert radiologists and might represent a valid and less invasive treatment for chest wall chondroid hamartoma, avoiding skeletal deformities.

## Introduction

Mesenchymal hamartoma is an extremely rare benign tumor, arising from the tissues of the chest wall. It is usually diagnosed during childhood and is frequently of congenital origin. Rarely symptomatic, it can cause either respiratory distress or chest wall mass, depending on its size and location. Although spontaneous regression has been reported, it commonly shows progressive growth.

Many authors have suggested en bloc excision as treatment of choice for mesenchymal hamartomas [1, 2], even though severe postoperative orthopedic problems may follow. Conservative management has been described, but experience in this field is limited, with few cases reported worldwide with relatively short-term follow-ups.

The aim of our study was to describe the results of percutaneous techniques, including radiofrequency thermoablation, microwave thermoablation and cryoablation, for the treatment of chest wall hamartomas in children.

## Materials and methods

We retrospectively collected data of children treated for mesenchymal hamartomas from 2005 to 2020 at our institution. Various percutaneous techniques were adopted: CT-guided radiofrequency thermoablation (VCT Tip RF needle [VER SAN, Verona, Italy] with 10–15 mm of exposed distal extremity and 12-min cycles at 80 °C), CT-guided microwave thermoablation (HC microwave thermoablation needle [SURGNOVA Healthcare Technologies, Zhejiang, China] with 7-min cycles at 45 W alternating with 5-min cycles at 75 W) and CT-guided cryoablation (IceRod, IceSphere or Ice FORCE needles [Boston Scientific, Milan, Italy], depending on the morphovolumetry of the lesions; with 2 cycles of 10 min reaching −20 °C to −40 °C core temperature and 5 min of thawing). We used SyngoVia software (Siemens Healthineers, Erlangen, Germany) to calculate three-dimensional (3-D) volume of each lesion at the time of diagnosis and after treatment based on CT or MRI follow-up.

The institutional review board determined that no ethical approval was required because of the retrospective and non-operative nature of the study.

## Results

Five children were treated for mesenchymal hamartomas of the chest at our institution from 2005 to 2020. Four children (80%) were boys and the mean age at diagnosis was 11.5 months (range 1–168.7 months). Only one child did not receive the diagnosis in the perinatal period. Two children (40%) presented with a palpable thoracic mass and three were incidentally diagnosed at chest radiography performed for dyspnea.

**Fig. 1:**
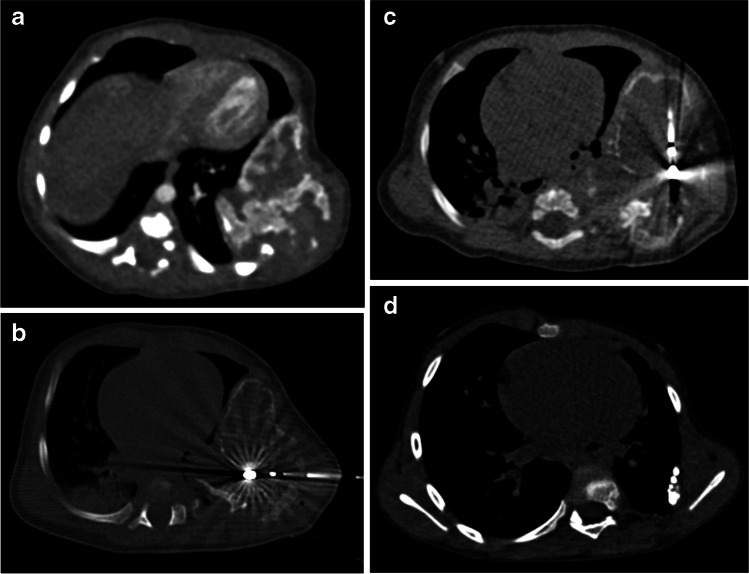
Mesenchymal hamartoma in a 2-week-old boy at diagnosis (patient 1) treated with microwave thermoablation. **a** Axial CT scan with contrast agent shows an inhomogeneously hypodense oval lesion involving the 5th–6th left ribs. **b, c** The boy underwent two microwave thermoablation sessions (axial CT images) followed by a dimensional increase in the mass, requiring surgical resection. **d** After 21-month follow-up, axial contrast-enhanced CT scan demonstrates almost complete resection of the lesion

**Fig. 2:**
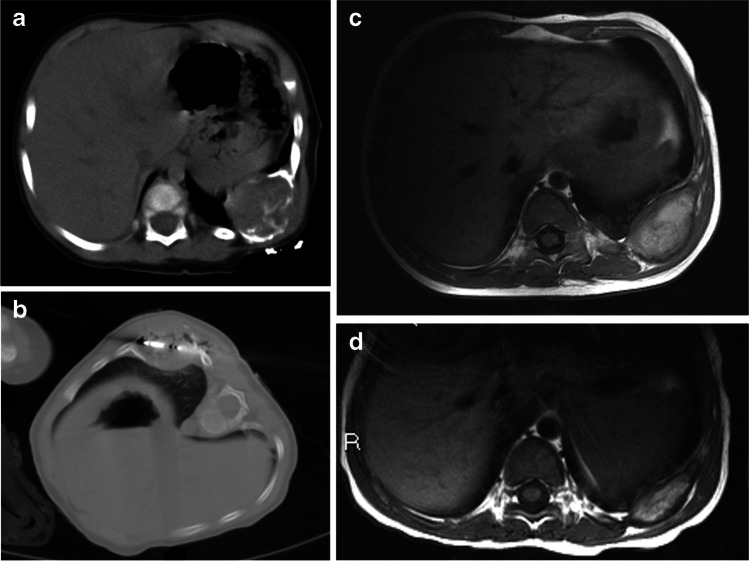
Mesenchymal hamartoma in an 11-month-old boy (patient 2) treated with microwave thermoablation. **a, b** He presented with a clinical mass of the 9th–10th left ribs, as confirmed on axial CT (with contrast agent) at diagnosis (**a**), and he underwent microwave thermoablation (axial CT, **b**). **c, d** Follow-up MRI. At first (1-month) follow-up, axial T1-weighted MRI (**c**) shows an inhomogeneous hyperintense lesion, which decreased in dimension on the 9-month follow-up axial T1-weighted MRI (**d**)

**Fig. 3:**
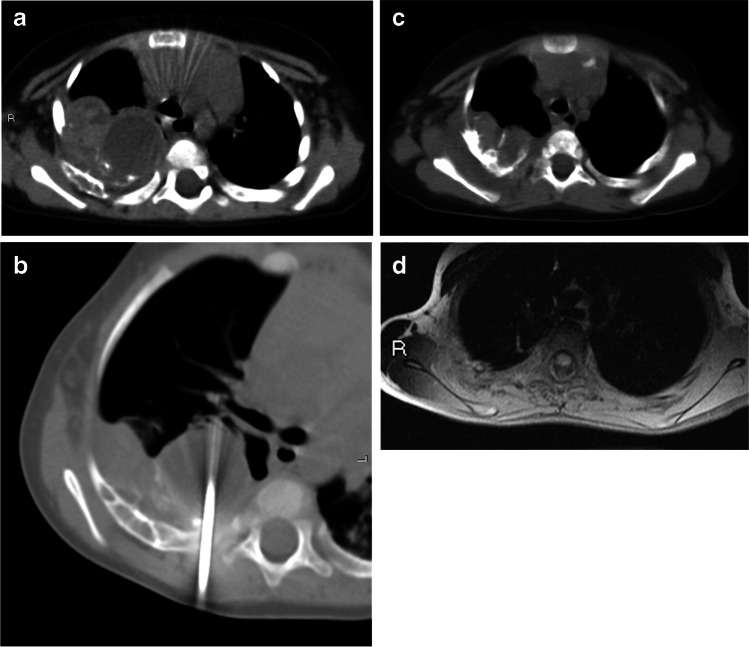
Mesenchymal hamartoma in a 6-month-old girl (patient 3) treated with radiofrequency thermoablation. She presented with persistent respiratory distress requiring diagnostic studies including chest radiograph and, successively, CT scan. **a** Axial CT shows a inhomogeneous oval mass arising from the 1st–2nd right ribs. **b** Two sessions of radiofrequency thermoablation were performed (axial CT). **c, d** Six-month follow-up images show a significant dimensional decrease of the tumor, as a smaller inhomogeneous mass with calcifications at axial CT (**c**) and as hypo/isointense residual disease on axial T1-weighted MRI (**d**)

The mean dimension of the neoplasia was 25.9 cm^3^ (range 7.6–119.0 cm^3^); one of them showed bilateral localization with dimensions 30.6 cm^3^ for the left side and 119.0 cm^3^ for the right side (Figs. 1, 2 and 3). All children underwent CT-guided biopsy to confirm the diagnosis (mesenchymal hamartoma).

Two children underwent microwave thermoablation, respectively, at 0.5 (Fig. 1) and 11 months of age (Fig. 2). The older boy, despite having a second microwave thermoablation procedure after 2 months, showed dimensional increase of the tumor (104.2 cm^3^ vs. 21.5 cm^3^ at diagnosis) and subsequently underwent surgical removal of the tumor.

Radiofrequency thermoablation was performed twice in one case (Fig. 3), at 11.5 and 14.5 months of age, for a 25.7 cm^3^ lesion of the 1st–2nd right ribs; the tumor showed 60% reduction, with a final dimension of 10.3 cm^3^.

**Fig. 4:**
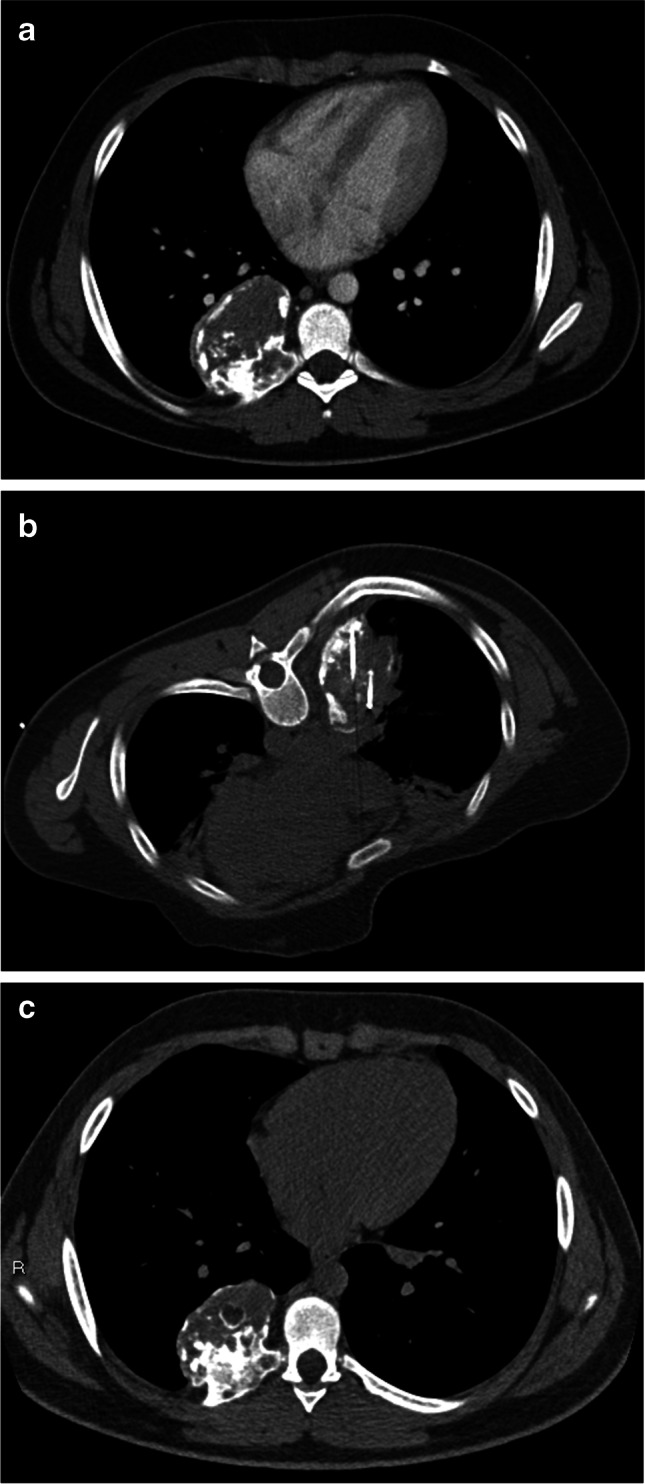
Incidental mesenchymal hamartoma in a 163-month-old boy (patient 4) treated with cryoablation. The mass, involving the 6th–7th–8th right ribs, was found at chest radiography. **a** Axial chest CT with contrast agent confirms the finding. **b, c** The boy underwent two sessions of cryoablation (intraoperative axial CT, **b**) with a slight dimensional increase in the lesion, as shown by the axial CT scan at last 18-month follow-up (**c**)

**Fig. 5:**
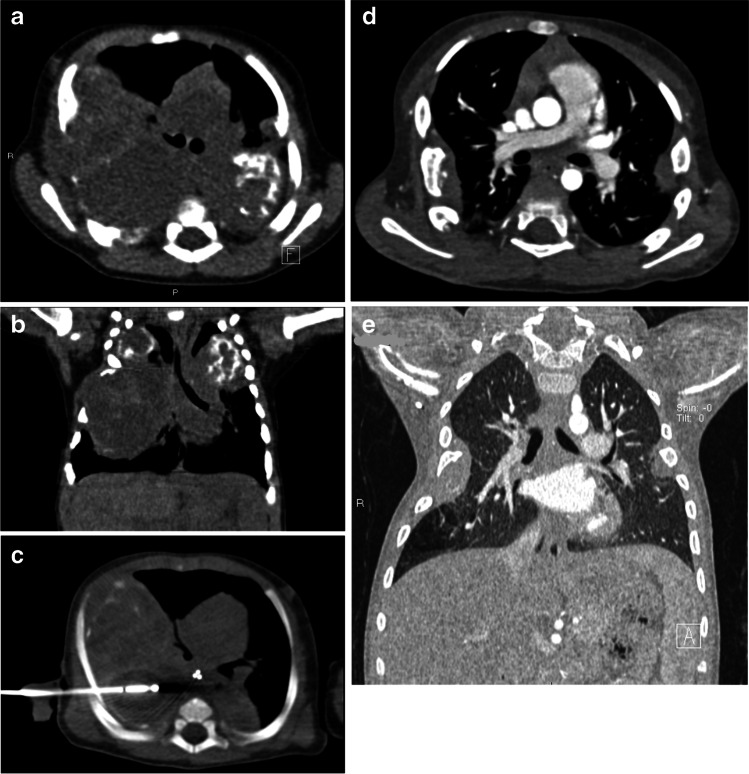
Mesenchymal hamartomas in a 4-month-old boy (patient 5) who was treated with cryoablation followed by radiofrequency thermoablation. He presented with acute respiratory distress and chest radiograph revealed bilateral masses of the thoracic wall. **a, b** Contrast-enhanced CT shows inhomogeneously hypodense oval lesions, with calcifications, arising from 4th–5th–6th right ribs and 4th left rib (**a,** axial; **b,** coronal). **c** He had cryoablation (axial CT image). Initial treatment was unsuccessful (tumor development and persistent dyspnea were seen), and he later underwent radiofrequency thermoablation. **d, e** Results were very good at 2-year follow-up, confirmed by contrast-enhanced axial (**d**) and coronal (**e**) CT images

Two children underwent cryoablation (Figs. 4 and 5). One of them (the child with bilateral localization of the mesenchymal hamartomas), subsequently underwent radiofrequency thermoablation because of tumor growth, with very good results at 2-year follow-up (96.5% of tumor reduction of the right lesion and 79.0% of the left).

The median reduction in lesion size was 69.6% (range 24.0–96.5%); one child, as described, showed dimensional increase of the tumor (385.1%) necessitating surgery. All children were discharged on the first postoperative day, except for 2 cases (patients 1 and 2) where pre-existing dyspnea remained. Postoperative pain was well tolerated and easily controlled with paracetamol (Table 1).

**Table 1:** Patient characteristics

Patient no.	Age at diagnosis (months)	Sex	Localization	Symptoms at diagnosis	Initial volume (cm^3^)	Technique	Number of procedures performed	Postoperative volume (cm^3^)	Percentage volume variation	Necessity for further procedure
1	0.5	M	5th–6th left ribs	Clinical mass	21.5	MWT	2	104.2	+385.1%	Surgical procedure
2	11	M	9th–10th left ribs	Clinical mass	7.6	MWT	1	5.8	−24.0%	N/A
3	6	F	1st–2nd right ribs	Respiratory distress	25.9	RFT	2	10.3	−60.3%	N/A
4	163	M	6th–7th-8th right ribs	Incidental diagnosis in chest radiograph	109.1	Cryoablation	2	122.4	+12.2%	N/A
5	4	M	4th–5th–6th right ribs, 4th left rib	Respiratory distress	Right 119.0 Left 30.6	Cryoablation + RFT	1 cryoablation + 1 RFT	Right 4.1 Left 6.4	−96.5% −79.0%	N/A

*F* female, *M* male, *MWT* microwave thermoablation, *N/A* not applicable, *no.* number, *RFT* radiofrequency thermoablation

## Discussion

Mesenchymal hamartomas are extremely rare benign tumors, with a reported incidence of 1 in 3,000 (0.03%) primary bone tumors [3, 4]. They usually occur within the medullary cavity or on the surface of ribs and can affect multiple ribs simultaneously [5]. They are typically solitary, but bilaterality or multicentricity occurs in a minority of cases. They are usually congenital lesions, diagnosed in childhood during investigations for respiratory distress or chest wall mass. The severity of the symptoms is related to the location and size of the lesion and ranges from mild cough to severe respiratory insufficiency.

Recommendations for treatment of chest wall hamartoma vary. Many authors have described treating most children by surgical excision [2], with a significant rate of complications, including hemorrhage and post-surgical scoliosis, especially if an en bloc excision is performed. As a result of these observations, conservative management and noninvasive treatment of mesenchymal hamartomas have been suggested, but few cases have been reported in the literature. Percutaneous techniques, recently developed, represent a valid alternative to surgery, considering their minimal invasiveness and widely reported advantages [6–8].

Radiofrequency thermoablation is a percutaneous technique performed under CT guidance that ablates parts of the pathological tissue. In radiofrequency thermoablation, an electrode connected to a generator is placed directly into the tumor tissue and a reference electrode (grounding pad) is placed on the child’s skin. When an electric current (frequency of radio waves 460–480 kHz) is applied, tissue heating results from resistive energy loss (frictional heating) as electrons agitate ionic molecules in tissue as they move toward the reference electrodes. The goal temperature is 60–100 °C, considered lethal to neoplastic tissue. Researchers described a “heat-sink effect” from vessels and airways that dissipate heat away from the normal adjacent tissue and concentrate the energy within the solid component of the target lesion [9, 10]. Radiofrequency thermoablation has been applied with success in osteoid osteoma and epiphyseal chondroblastoma, and we decided to use the same technique to treat chondroid hamartomas because of their similarity in terms of histology [11]. The greatest advantage of radiofrequency thermoablation is experience, because it represents the most diffuse and established noninvasive technique for unresectable tumors, in both adults and children [9]; however, it cannot be applied in the mediastinum or high in the lung apex because mechanical and thermal injuries after radiofrequency thermoablation have been reported [10]. In our study, one child underwent CT-guided radiofrequency thermoablation only and one child, who had severe respiratory distress as first symptom at diagnosis, had radiofrequency thermoablation treatment following one CT-guided cryoablation treatment.

Authors have recently reported the use of microwave thermoablation for neoplastic lesions, like liver and pulmonary metastases in adults because of the lack of current through the tissues and the heat-sink effect [12]. Indeed, electromagnetic microwaves traveling at 9.2×108 Hz cause friction and heat by agitating water molecules within the surrounding tissues, leading to coagulation necrosis and cell death [13]. Compared with radiofrequency thermoablation, microwave thermoablation shows improved penetration, minor dehydration and heating effects, and consistently higher intra-tumoral temperature, making this technique more suitable for lesions larger than 5 cm and cystic lesions [9, 14]. At our institution, two children underwent CT-guided microwave thermoablation. The first child was a 0.5-month-old boy at diagnosis; he had a mass localization at the 5th–6th left ribs and a clinically palpable mass as the first symptom at diagnosis. The second child was also male, 11 months old at diagnosis with mass localization at the 9th–10th left ribs, also with a clinically palpable mass as the first symptom at diagnosis; this child experienced a decrease in mass size. The first child, however, underwent surgery following microwave thermoablation because of an increase in mass volume.

Cryoablation is a percutaneous technique based on the “Joule-Thomson effect,” in which gas in an area of high pressure travels to a region of lower pressure, allowing the gas to expand and become cooler. In vivo, it causes direct injury to cellular membranes and vascular endothelium through the alternating formation and thawing of intracellular ice [9, 15]. Furthermore, the resulting endothelial damage leads to platelet aggregation and microthrombosis. Cryoablation represents an established treatment for both symptom management and local control of benign and malignant neoplasia, especially in adults [16–18]. Its advantages, compared to radiofrequency thermoablation, include larger tumor ablation volumes, the possibility of using multiple applicators and less procedural pain [9]. In our study, two children were treated with cryoablation; one of them also underwent radiofrequency thermoablation because of an increase in tumor volume after the first cryoablation procedure. The child treated exclusively with CT-guided cryoablation, a 163-month-old boy at diagnosis with mass localization at the 6th–7th–8th right ribs with incidental diagnosis of the mass by chest radiograph, showed a non-regression of mass volume. The reason for this failed outcome could be related to the higher age of the child at first treatment and a different reaction of tumoral cells to cryoablation, but further studies, especially regarding molecular and cellular response to thermic injury, are mandatory to confirm our theory.

## Conclusion

As demonstrated by our experience, image-guided tumor ablation can be successfully performed using various techniques of controlled, direct thermal (heating or cooling) destruction of cells and tissue in an established procedure, resulting in significant volume reduction. Being minimally invasive, these procedures are associated with reduced morbidity and mortality, conservation of normal lung tissue and lower procedural cost. Furthermore, these techniques seem to be well tolerated in the pediatric age and avoid the risk of postoperative progressive skeletal deformity.
